# Recycling bread waste into chemical building blocks using a circular biorefining approach

**DOI:** 10.1039/d1se00575h

**Published:** 2021-09-06

**Authors:** Vivek Narisetty, Rylan Cox, Nicholas Willoughby, Emel Aktas, Brijesh Tiwari, Avtar Singh Matharu, Konstantinos Salonitis, Vinod Kumar

**Affiliations:** School of Water, Energy and Environment, Cranfield University Cranfield MK43 0AL UK Vinod.Kumar@cranfield.ac.uk +44 (0)1234754786; School of Aerospace, Transport and Manufacturing, Cranfield University Cranfield MK43 0AL UK; Institute of Biological Chemistry, Biophysics and Bioengineering, School of Engineering and Physical Sciences, Heriot-Watt University Edinburgh EH14 4AS UK; School of Management, Cranfield University Cranfield MK43 0AL UK; Teagasc Food Research Centre Dublin D15 KN3K Ireland; Green Chemistry Centre of Excellence, University of York, Department of Chemistry Heslington York YO10 5DD UK

## Abstract

Food waste is a global problem, causing significant environmental harm and resulting in substantial economic losses globally. Bread is the commonly wasted food item in the developed world and presents a severe problem for the majority of European nations. It is the second most wasted food item in the UK after potatoes, with an equivalent of 20 million slices of bread thrown away daily. Bread is a starchy material and a rich and clean source of easily extractable fermentable sugars – this is in direct contrast to lignocellulosic feedstocks where harsh physical, chemical and/or enzymatic pretreatment processes are required for release of fermentable sugars. Furthermore, these necessary lignocellulosic pretreatment methods often produce sugars contaminated with fermentation inhibitors. Therefore, bread waste presents a clear opportunity as a potential carbon source for novel commercial processes and, to this end, several alternative routes have been developed to utilize bread waste. Possibilities for direct recycling of bread waste within the food industry are limited due to the relatively short material lifetime, stringent process and hygiene requirements. Anaerobic digestion (AD) and incineration are commonly employed methods for the valorisation of bread waste, generating limited amounts of green energy but with little other environmental or economic benefits. Most food wastes and by-products in the UK including bakery waste are treated through AD processes that fail to harness the full potential of the these wastes. This short communication reviews the challenges of handling bread waste, with a focus on a specific UK scenario. The review will consider how bread waste is generated across the supply chain, current practices to deal with the waste and logistics challenges in waste collection. The presence of clean and high-quality fermentable sugars, proteins and other nutrients in bread make it an ideal substrate for generating chemicals, fuels, bioplastics, pharmaceuticals and other renewable products through microbial fermentations. We suggest potential applications for recycling bread waste into its chemical building blocks through a fermentative route where a circular biorefining approach could maximize resource recovery and environmental savings and eliminate waste to as close to zero as possible.

## Abstract 2

Food waste is a global problem, causing significant environmental harm and resulting in substantial economic losses globally.

## Graphical abstract

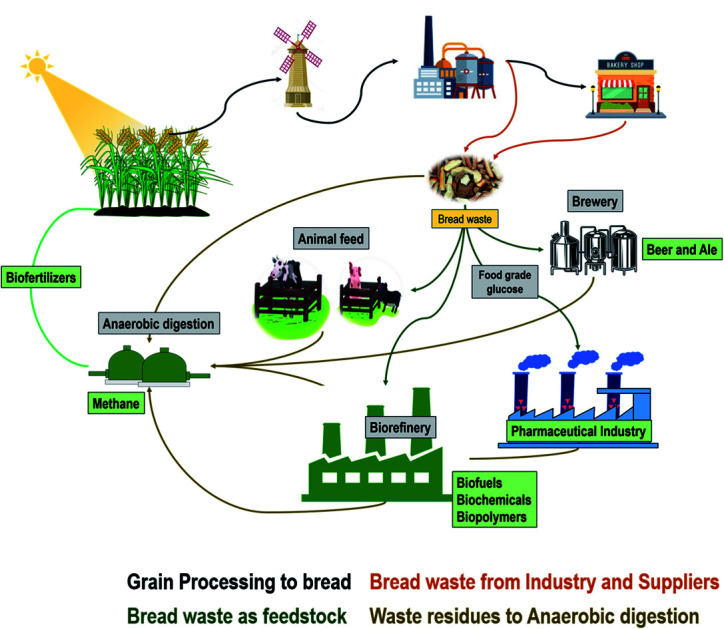

## Bread wastage – a serious global problem

In nature, there are a number of iconic and irresistible fragrances such as scent from gasoline, a new car, a new notebook, the soil after fresh rainfall, and the sweet aroma from a bakery. When we pass a bakery where fresh bread is being baked, we can sense the sweet aroma filling the air around the bakery. This sensation is lost with wrapped or packed bread, which is widely available in supermarkets. Bread is a universal staple across most income ranges, varying in cost from £0.30 to £20 for a loaf, based on the quality of ingredients and the marketing strategy of the baker. Bread is sold and consumed across the entire social and geographical spectrum. Since bread can be a relatively cheap or an expensive luxury depending on ingredients, the production process and marketing, hence consumption and therefore wastage are likely to be ubiquitous globally. Bread is a commonly wasted food in a majority of countries in the developed world and is a particularly serious problem in most European nations. The global annual production of bread is >100 million tons. According to global bread market split analysis, Europe dominates the market with a share of 53.6%, followed by the US (28.6%), Asia Pacific (10.9%) and Middle East and African countries (6.9%) [World Bread Market and Trends||BBM Magazine (http://magazinebbm.com)]. It is difficult to quantify the precise amount of wasted bread, but it has been approximated that, globally, ∼10% of all manufactured bread is wasted.^1^ The bread loss does not simply reflect the product loss, but the loss of various natural resources such as water, land, and energy used for the production of raw materials, transportation, and manufacturing. Even the bread and the other materials lost during these processes have a significant impact on society, the environment, and the economy.^2^ In the Netherlands, the volume of bread wasted accounts for an economic loss >400 million euros. (Factsheet-Bread-Waste.pdf (foodwin.org)). The situation is significantly worse in developed economies, and, in the UK, bread is the second most wasted food with as much as 44% of manufactured bread going to waste, causing massive economic loss and environmental concerns [How Much Bread Do We Waste in the UK? (ecoandbeyond.co)]. Every day, about 20 million slices of bread are thrown away in the UK, leading to an annual wastage of 292 000 tons, which corresponds to 584 000 tons of CO_2_ equivalent emissions.^3^ Unless modern techniques such as anaerobic digestion (AD) is adapted for food waste management, in most of the developing countries, bread or food wastes end up in landfills due to limited resources and infrastructure. As an organic biogenic waste, bread waste could cause serious risks of public and environmental issues with respect to health, and pollution of natural habitats.^4^ Therefore understanding the process of waste generation and holistic approaches to tackle these wastes at various steps in the supply chain might assist in building up the economy either by reducing the wastage or recycling waste into valuable products.

## Manufacturer to the customer: process of waste generation

Bread disposed of during any supply chain stage is considered bread waste, an entirely avoidable waste as bread has no inedible fraction. In the bakery industry, it is crucial to understand the process of waste generation which arises in different stages of the supply chain from the manufacturer to consumer or production to distribution to prevent wastage of bread and thus improve the process economics as well as sustainability. Improved knowledge in this area would allow the development of effective measures to limit or prevent waste generation. Goryńska-Goldmann *et al.* (2021) identified the following stages as possible retrieval points for bread waste in the bakery sector: making and handling intermediate products and dough; portioning and forming a dough, baking, customised packing, shipping (storage), and transport by own fleet.^5^ Bread wastage during the manufacturing stage can occur due to substandard practices, processing factors, faults during the operation, rejection during the quality control of the product, improper handling during the storage/packing and sometimes due to the type of product manufactured. For example, up to 40% of bread is lost in the sandwich-making process due to the removal of crusts from loaves.^6^ Later the packed bread finds its way to retail stores or supermarkets through distributers. Storage and transport conditions play an essential role in maintaining the bread in a healthy environment to ensure high quality and a good lifespan. Bread is a staple food produced for human consumption and considered waste if, due to any reasons, it is not consumed or becomes unfit for human consumption.^7^ In the UK, a large amount of bread (32%) is wasted at the consumer level, and one of the primary reasons for this is its limited shelf life.^8^ People's lack of awareness exacerbates this situation as they purchase more than they need and have insufficient knowledge on storage conditions and the shelf life. This scenario is not unique to the UK, and similar problems are observed in many European countries. Stensgård and Hanssen (2016) and Brancoli *et al.* (2019) identified bread as a significant waste generated in Norway and Sweden.^7,9^ Bread waste generated at any stage of the supply chain causes enormous loss of potential nutrients and significantly impacts the carbon footprint of the process. Furthermore, such wastage has a knock-on effect in terms of wastage of energy resources invested in wheat production, post-harvesting, manufacturing of bread, transport, and storage. For example, one-fifth of greenhouse gas (GHG) emissions in the UK associated with food and drinks are generated during their production and distribution, so any wastage in these industries has clear negative environmental and societal impacts (Our waste, our resources: a strategy for England (http://publishing.service.gov.uk)). A service scheme take-back agreement (TBA), implemented in most European countries, makes the bakery responsible for any unsold products. Therefore, they must arrange collection and treatment by reversing the supply chain. Bakeries under the TBA are financially accountable for unpurchased products, and their collection and treatment. Hence, the bakers must forecast the amount to be ordered by the retailers and manage the product shelf-life to reduce waste.^7,10^ Process improvements and technological advancements can minimize the amount of waste generated.

Better insights into the ingredient's quality, quantity, shelf-life and monitoring would also reduce wastage in manufacturing, as would clearer understanding of the bottlenecks in storage and transportation. “Just-in-time” manufacturing strategies to match requirement based on a more detailed understanding of the demand and logistics would also be advantageous in waste reduction. Brancoli *et al.* (2019) considered the role of sales, the pack-size, the shelf-life and the take-back agreement (TBA) on the loss rate, and the work demonstrated significant differences in wastage due to the TBA, *i.e.*, the type of distribution system has a considerable impact on the amount of wastage and TBA liable bread tends to be wasted more than non-TBA products.^7^

## Standard practices of bread waste management

The accurate forecasting of demand is a complex task as it is influenced by multiple factors. In addition, consumers always prefer to buy fresh bread with a long shelf-life (3–5 days); therefore, bread with a short expiry date (1–2 days) is more likely to be wasted. Hence, bread wastage is inevitable within the supply chain – it can be minimised but cannot be eliminated. It is essential to develop methods to cut down the surplus of bread generated in the first place as waste costs money, time and resources that can be utilized elsewhere. A clear challenge, therefore, is how to address this problem of solid waste management. One solution to reduce waste could be to provide better knowledge and information to consumers to deal with their food-related actions from an economic and environmental standpoint. If waste cannot be further reduced, possible solutions to reutilize bread waste include supplementation as animal feed,^11^ incineration,^12^ anaerobic digestion,^4^ and a carbon source for beer and ethanol production^7,10^ should be considered. Bread wastage is broadly concentrated at domestic and supplier-retailer levels, but with a clear difference. At the supplier-retailer level, bread waste is not contaminated with other food wastes, can be managed more easily and is more suitable for valorisation.^5^ Bread wastage in households is mixed with other food waste or municipal solid waste and therefore requires segregation for specific processing. The main obstacle for efficient utilisation of household bread waste is therefore the need to introduce separate collection bins to segregate the bread from other wastes, which would add cost and complexity of operation to the process.^13^ Eriksson *et al.* (2015) performed a life cycle assessment (LCA) study for six waste management scenarios (landfill, incineration, composting, anaerobic digestion, animal feed and donations), using five different food products (bananas, grilled chicken, lettuce, beef and bread) and found that better management of bread waste had the most significant potential for reducing GHG emissions.^14^ Recently, Brancoli *et al.* (2020) conducted an LCA of the management of surplus bread, and the different management options investigated were as food donation, use as animal feed, beer production, ethanol production, anaerobic digestion (AD), and incineration.^10^ Reduction of bread waste at the source had the highest environmental benefit, followed by feed production, donation, beer production and ethanol production without a clear preference between these four latter valorisation pathways. AD and incineration are observed with the lowest environmental savings. AD also suffers from a significant amount of loss of bread waste (∼44%) during pre-treatment, though loss can vary between plants depending on parameters.^15^ In the UK, most food waste, including that from bakeries, goes to AD and, to a lesser extent, incineration, composting, and landfilling. A shift from AD to bioconversion of bread waste into biofuels like ethanol, can enhance the environmental savings of 0.56 kg CO_2_ eq. kg^−1^ bread waste, which is equivalent to 163 520 tonnes of CO_2_ per year (considering 292 000 tonnes of bread waste per year). Even the conversion into animal feed could reduce 1549 kg CO_2_ eq. per year.^10,15^

AD is associated with low environmental savings and is not the most suitable technology to harness the full potential of surplus bread, a rich source of food-grade glucose. Recently, some innovative work to valorise bread waste has been started. For example, some breweries in the UK have started using bread waste to substitute malted barley to act as a source of sugar for fermentation in beer manufacturing. In 2017, Greencore collaborated with Toast Ale to provide bread waste from sandwich production to the Adnams brewery, where 25–28% of original malt was replaced with dried bread.^10,16,17^ Bute Brew Co. in Scotland proposed a similar approach in 2018, with unsold bread replacing between 20 and 25% of the malted barley used in the production of 5.1% alcohol crafted beer Thoroughbread.^18^ In both these cases, the proportion of malt that can be replaced is strictly limited, as the barley contains natural enzymes that can break down bread starch to fermentable sugars, meaning replacing more than around 25% of the malt is impractical. As the amount of malted barley decreases, supplementation of external enzymes for gelatinization and saccharification increases. In a study conducted by Immonen and associates, to recycle the waste bread for production of fresh wheat bread, they observed that bread waste processed or fermented with lactic acid bacteria (*Weissella confusa* A16), provided better softness and viscosity to the dough in comparison to the untreated bread waste. Further to fermentation, lactic acid bacteria produced dextran or β-glucan exopolysaccharides, and yielded residual glucose or fructose. Therefore, no extra sugar supplementation was required for yeast leavening. Further fermentation with LAB strains increased the acidic nature of the bread, thus improving hygiene.^19^ Efforts have also been initiated to use glucose from bread waste for manufacturing pharmaceutical molecules. GlaxoSmithKline (GSK), Veolia and the Biorenewables Development Centre, York are collaboratively working on a project to investigate the production of one of GSK's pharmaceutical active compounds from bread by-products instead of directly from wheat.^20^

## Holistic approaches for bread waste valorisation *via* bioconversion

The UK has 500+ active AD plants, of which 79 treat food waste, while there are only four bioethanol plants and in which two are in operation.^25^ The logistics and local infrastructure for AD treatment of bread waste are already in place as AD plants are present in all over the UK, reducing the transportation cost.^21,22^ Nevertheless, to maximize the potential of bread waste and improve environmental performance, focus should be placed on encouraging the development of biomanufacturing hubs for fermentative production of fuels and chemical building blocks, including platform, fine, speciality and commodity chemicals.^20^

**Fig. 1:**
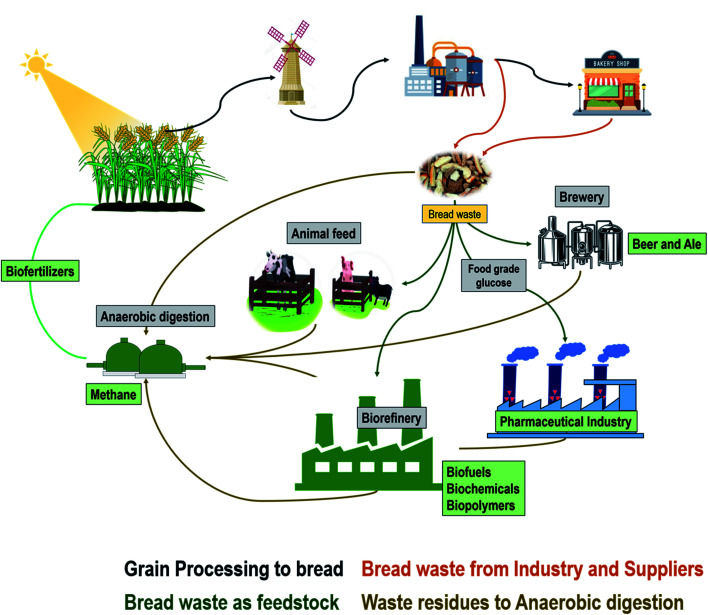
Multiple routes for recycling of bread waste.

First-generation biorefineries using edible feedstocks such as corn, sugarcane, vegetable oils *etc.* for fuels and chemicals through fermentative routes have been quite successful and generate a billion litres of biofuels every year. However, the primary concern with an exponentially growing human population is that if edible raw materials are utilized to produce fuels & chemicals, then there may be a shortage of food commodities to fulfil the demand of the global population.^23,24^ To avoid the food *versus* fuel/resource debate, the focus of biorefining studies has generally been diverted towards non-edible feedstocks. However, most non-edible feedstocks require energy-intensive harsh pre-treatments and are associated with impurities and inhibitors such as organic acids, furan, and lignin derivatives. As an alternative, bread waste is a starchy material and a clean source of fermentable sugars and proteins. Typically 100 g of bread contains around 50–70 g carbohydrate, 8–10 g protein, 1–5 g fat and traces of phosphorus.^25^ Sugars and amino acids can be obtained from bread *via* enzymatic hydrolysis, which has several outstanding advantages including mild reaction conditions, avoidance of toxic chemical usage and minimal/no risks of generation of fermentation inhibitors.^26^ Furthermore, the composition of bread/bakery waste is consistent and homogeneous and the obtained sugars are as good as pure sugars. All of these reasons make bread waste an attractive potential feedstock for microbial fermentative production of a broad range of products, including industrially essential chemicals and fuels with high market values (Fig. 1).^27^ Sugar hydrolysates from bread waste are generally devoid of growth inhibitors and can be a suitable medium for the growth of the majority of microbial chassis strains with commercial potential to produce high value-added chemicals and fuels such as 2-keto-d-gluconic acid,^28^ lactic acid,^29^ succinic acid,^26^ pigments,^30^ aromatic compounds,^31^ and ethanol.^32^ Currently, most of these chemicals are manufactured through a petrochemical-based route and are therefore associated with adverse environmental performance. On the other hand, if bread waste-based sugar was employed for fermentative production of chemical building blocks, it will result in an economical process and contribute towards a more sustainable environment.

Table 1 summarises the fermentative production of high-value products, including fuels, chemicals, enzymes and edible materials from bread waste in the last 5–10 years. Leung and associates (2012) used bread waste for fermentative production of succinic acid (SA), a top platform chemical, and observed an accumulation of 47.3 g L^−1^ SA with a yield of 0.55 g SA per g bread.^26^ This was followed by the work of Gadkari *et al.*, who carried out a LCA of the bioprocess and found a better environmental profile and significantly lower NREU (non-eenewable energy units) in comparison to fossil-based SA production.^33^ Sadaf and associates employed different modes of fermentation using bread waste containing 598 mg g^−1^ reducing sugars for lactic acid (LA) production by several lactic acid bacteria. Like SA, LA is another platform chemical as per the revised list by the US Department of Energy. The three different strains of *Lactobacillus paracasei* SKL-9, SKL-11 and SKL-21 could produce 26.4, 28, and 27 g L^−1^ LA *via* simultaneous saccharification and fermentation with conversion yields of 53, 56, and 54 mg LA per g bread waste, respectively. In the case of solid state fermentation, LA accumulated by SKL-9, SKL-11 and SKL-21 was 212, 223, and 250 mg LA per g bread waste respectively.^29^ The latest published work by Maina *et al.* (2021) demonstrates the production of 2,3-butanediol (BDO) and acetoin, two commercially important chemicals with huge significant market potential, from bread waste hydrolysate by *Bacillus amyloliquefaciens*. The fed-batch culture with bread hydrolysate at a *k*_L_*a* (volumetric oxygen transfer coefficient) of 110 h^−1^ resulted in a mixture of acetoin and of meso- and d-isomers of BDO with a total concentration of 103.9 g L^−1^ while at higher *k*_L_*a* (200 h^−1^) there was a shift in metabolism and acetoin (65.9 g L^−1^) was the main product.^34^ In a recent study, Torabi *et al.* (2020), employed bread waste as a feedstock for bioethanol production by *Saccharomyces cerevisieae*. They reported high glucose yield from acid as well as enzymatic hydrolysis of bread waste. The ethanol yields achieved using glucose obtained from acid and enzymatic hydrolysis were 248 and 313 g per kg of dry bread residues.^35^ Like ethanol, hydrogen (H_2_) is also an efficient cleaner biofuel and can be produced through electrochemical, thermochemical and biological processes. In a study Han and associates (2017) saccharified bread waste through enzymatic hydrolysis and utilized the hydrolysate to produce biohydrogen. In this process 103 mL H_2_ per g bread waste was produced by *Biohydrogenbacterium* R3 through a two stage saccharification and dark fermentation strategy.^36^

**Table 1:** Microbial production of fuels, chemicals and enzymes using bread waste as a feedstock

S. no.	Feedstock	Microorganism	Product	Fermentation mode	Titer	Yield^a^	Productivity	Reference
1	Waste bread hydrolysate	*Biohydrogenbacterium* R3	Hydrogen	Separate hydrolysis and fermentation (SHF)	7482 mL	103 mL g^−1^	103.91 mL h^−1^	36
2	Waste bread hydrolysate	*Saccharomyces cerevisiae*	Ethanol	SHF	58 g L^−1^	0.35 g g^−1^	1.21 g L^−1^ h^−1^	32
3	Waste bread hydrolysate	*Saccharomyces cerevisiae*	Ethanol	SHF	33.9 g L^−1^	0.25 g g^−1^	0.36 g L^−1^ h^−1^	35
4	Waste bread hydrolysate	*Saccharomyces cerevisiae*	Ethanol	SHF	100 g L^−1^	0.35 g g^−1^	10 g L^−1^ h^−1^	45
5	Waste bread	*Lactobacillus paracasei*	Lactic acid	Simultaneous saccharification and SSF (solid-state fermentation)	28 g L^−1^	0.056 g g^−1^	0.58 g L^−1^ h^−1^	29
6	Waste bread hydrolysate	*Actinobacillus succinogenes*	Succinic acid	SHF	47.3 g L^−1^	0.55 g g^−1^	1.12 g L^−1^ h^−1^	26
7	Bread crumbs	*Thraustochytrium* sp. AH-2	Lipids	Submerged fermentation	390 mg L^−1^	0.03 g g^−1^	2.32 mg L^−1^ h^−1^	46
8	Waste bread hydrolysate	*Bacillis amyloiquefaciens*	BDO + acetoin	SHF	103.9 g L^−1^	0.39^b^ g g^−1^	0.87 g L^−1^ h^−1^	34
9	Waste bread	*Rhizopus oryzae*	α-Amylase	SSF	—	100 units per g	—	38
10	Waste bread	*Rhizopus oryzae*	Protease	SSF	—	2400 units per g	—	38
11	Waste bread	*Aspergillus awamori* 2B.361 U2/1	Glucoamylase	SSF	—	114 units per g	—	27
12	Waste bread	*Aspergillus awamori* 2B.361 U2/1	Protease	SSF	—	83.2 units per g	—	27
13	Waste bread	Enzymatic hydrolysis (α-amylase + glucoamylase) + biotransformation (glucose isomerase)	Glucose–fructose syrup	Sequential hydrolysis and enzymatic biotransformation	—	0.45 g g^−1^ (glucose) + 0.4 g g^−1^ (fructose)	—	47
14	Waste bread hydrolysate	*Aspergillus* sp.	Protease	Submerged fermentation	—	117 units per g	—	39
15	Waste bread hydrolysate	*Aspergillus sp.*	Glucoamylase	Submerged fermentation	—	8 units per g	—	39

a Yield: calculated per gram of waste bread saccharified for glucose production.

b Yield calculated per gram glucose.

Bread waste has been recycled for manufacturing edible products. There are examples where breweries have started to utilize unused bread waste in the beer brewing process.^17,18^ For example, Jaw Brewery Limited in Scotland uses bread waste from Thomas Auld and sons Ltd (Aulds bakery) to create low-alcohol beer.^18^ In another study, Gmoser *et al.* (2020) enhanced the value of stale bread by transforming it into a nutrient enriched product through solid state fermentation using edible filamentous fungi *Neurospora intermedia* and *Rhizopus oryzae*. The protein content increased from 16.5% in stale sourdough bread to 21.1% in the final fermented product with a improved amino acid profile. In addition, an increment in dietary fiber, minerals (Cu, Fe, and Zn), α-linolenic acid, vitamin E and ergocalciferol (D2) was noticed.^37^ Besides biochemicals and biofuels, industrial enzymes such as α-amylase, glucoamylase, and proteases have also been accumulated on bread waste *via* solid state fermentation. Benabda and Associates (2019) used fungus *Rhizopus oryzae* to produce hydrolytic enzymes α-amylase (100 U g^−1^) and protease (2400 U g^−1^) from bread waste.^38^ Similarly, Haque *et al.* (2016) made use of filamentous fungus *Monascus purpureus* to generate hydrolytic enzymes glucoamylase (8 U g^−1^) and protease (117 U g^−1^) from bread waste.^39^

Ethanol has several interesting properties as a biofuel and offers a number of advantages, such as a high-octane number, high heat vaporization, and combustion efficiency. In addition, bioethanol is less toxic and readily biodegradable and contains negligible amounts of sulphur.^40^ Currently, it is blended with petrol at up to 10% levels in many countries, including the UK. The demand for ethanol has also increased during the current COVID19 pandemic due to its application as a sanitiser and disinfectant. Recently, the UK Government has decided to ban all new petrol and diesel-based cars from 2030 to cut down carbon emission to meet the zero-emission goal by 2050. Bioethanol production uses energy from only renewable sources and contributes towards a carbon-neutral environment. It is anticipated that the demand will go further up in future because bioethanol is a low carbon fuel and can play a key role in decarbonisation of the transport sector. Therefore, it can make a significant contribution to achieving the renewable energy requirements and zero emission targets of the UK by 2050. However, ethanol production is currently not promising in the UK. Bioethanol production started in 2007 in the UK and increased from 29 million litres in 2011 to 645 million litres in 2017,^41^ but has plummeted in recent years, with plants regularly decommissioned for extended periods. All the ethanol in the UK comes from edible feedstocks such as sugar beet and wheat. The actual UK bioethanol production has generally been significantly lower than production capacity. Some of the bioethanol plants in the UK have ceased production for prolonged periods (Crop Energies, Teesside and Vivergo, Hull) due to low prices elsewhere, leading to high levels of importation of bioethanol. These two production plants have a production capacity of 820 million litres, constituting about 85% of the UK's installed bioethanol production capacity.^42^ Long-term sustainability of bioethanol production in the UK can be achieved through utilization of waste biomass rich in renewable and fermentable carbon such as bread waste and can also help in mitigating environmental impacts and align with the EU policy.^43^ There have been a few studies where ethanol has been generated using bread waste as feedstock, and an ethanol yield of ∼350 g per kg of bread has been achieved.^32^ Based on this, around 102 200 tonnes or 129.5 million litres of ethanol can be manufactured from annual UK bread waste and this would meet 15–20% annual demand for bioethanol in the UK. This will not only solve the problem of bread waste management but also generate extra revenues for bakeries and make the UK independent in terms of bioethanol production. Moreover, bread waste can also be utilized to produce other platform and fine chemicals, fuels and enzymes (Table 1), and the technology can be implemented to different kinds of similar single-source wastes. Thus, we propose recycling bread waste into chemical building blocks or ethanol through fermentative routes with a circular biorefining approach to maximize resource recovery, and environmental savings and eliminate waste to zero levels.

Currently, diverse microorganisms like bacteria (*Actinobacillus succinogenes*),^26^ yeast (*Saccharomyces cerevisiae*),^32,35^ and fungi (*Aspergillus terreus*)^44^ have been employed as microbial cell factories for bioconversion of starchy feedstocks like food, bakery and bread wastes into valuable products such as succinic acid, ethanol, and itaconic acid. Further, rational and process intensification approaches can be implemented to sustain the economic feasibility. Understanding the efficiency of bread waste as a sustainable feedstock, Dr Vinod Kumar’s group at the School of Water, Energy and Environment, Cranfield University, United Kingdom, is developing a circular bioeconomy approach towards valorising waste bread for the production of high value-added chemicals and fuels.

## Logistical challenges and changes for implication

As described in the previous section, utilisation of bread waste as an economical feedstock is a viable option that can drive costs down for the production of green chemicals. Many different renewable feedstocks have been investigated against their pure counterparts in the production of materials but what hasn't been covered in as much detail is the logistical and environmental impact of using waste. The concise useable life of materials such as bread waste makes the redistribution challenging. Waste materials must be quickly transported to the site of fermentation, ready for pre-processing and eventual fermentation. When considering the quantities required, especially at a commercial scale, this can result in tonnes of waste needing to be delivered and processed within a short period. If this is left unprocessed or the supply exceeds the production demand, then there is a possibility of food decaying and beginning to create an environmental health hazard to the workers, attracting rodents as well as degrading the material to such an extent that it is no longer suitable for use as a feedstock. Regulation of the quality of waste material and transportation timings must be carefully controlled to enable swift utilisation of the waste. There are many bread waste locations such as bakeries, supermarkets and domestic waste collection sites, where the distribution or supply chain remains an unsolved issue. In the TBA, a reverse flow of logistics flow enables the clean flow of bread waste, which is not possible at the domestic level where it is a part of the municipal solid waste. This contamination with other food wastes is the major obstacle for domestic bread waste as a resource, presenting challenges for separation. In addition, lack of control of timescales means that domestic bread waste, even if properly separated, may have begun to degrade to an unusable state before collection, resulting in issues in processing. Domestic collection would therefore likely require separated food waste bins requiring individual collections at short time intervals – a major feasibility challenge both from the perspective of household acceptability, and increased resource load on local councils. An alternative may be the provision of segregated single collections for domestic food waste to households, for example cotton bags/small bins for bread waste, which could form a part of pre-existing domestic food waste collection with separation taking place at biowaste collection plants. However this would not overcome timescale and degradation challenges, so supermarkets and bakeries still remain more viable options, although these have logistical issues too. Collection of bread waste from each bakery or supermarket would have to be introduced. Many supermarkets, for example, Tesco, already use their bread waste in-house for items such as sourdough and many others are signed up to agreements to return any unused waste to the manufacturer. Others already ship them to specific companies to deal with in a collective food waste process rather than focus on separation. It would require close partnership with the company's bakeries and supermarkets to implement a regular collection service solely for bread waste.

One final word of caution when using bread waste as the feedstock would be to consider the balance of GHG emissions through a complete life cycle analysis (LCA). Whilst it is the case that the use of waste as a feedstock diminishes the waste deposition at landfill sites and therefore, a reduction in amount the GHG emissions. But accepting the TBA agreement results in additional transportation for the collection of bread waste which may further contribute to increased GHG emissions. Of course, regardless of the disposal route, bread waste must likely be transported, but careful attention must be paid to the levels of transportation required for different processing routes. The use of bread waste for fermentation must be carefully monitored, and since specific processes such as ethanol fermentation produce carbon dioxide as a by-product, care must be taken to not add to the process carbon footprint though this CO_2_ production. Although fermentative CO_2_ is relatively pure, however, it could be relatively quickly contained and captured from the process. Throughout the world, various research groups in academia and industry are investigating technologies that can absorb CO_2_ from the atmosphere and either store it long term, use it directly in products such as carbonated beverages or convert it to long-chain alcohols and alkenes, which constitutes synthetic fuels. Hence bread waste can be a suitable and sustainable feedstock to produce value-added chemicals, biofuels, and synthetic fuels.

## Conclusions

Bread waste is potentially one of the major bioresources in the UK and, whilst wastage should be minimised, eliminating wastage completely from the bread supply chain is an unrealistic goal. Bread waste represents a substantial untapped rich source of clean and fermentable sugars and, to a lesser extent, proteins. It can be converted into high-value chemicals, biofuels, bioplastics and other biorenewable products with applications across many industries. Currently, the majority of bread waste goes for anaerobic digestion, but the potential exists for much higher value uses, and the scope of opportunity is enormous. The full potential of bread waste needs to be unleashed through using it as a feedstock for biomanufacturing of food and non-food products through fermentative routes. As discussed, Europe has greater opportunity and need for assessing the valorisation of bread waste as business case followed by US, and Asia Pacific countries. Based on the availability, and valorisation options, a business plan considering the bread waste as raw material for high value-added chemicals, biofuels, pharmaceutical products, beer and/or pet food ingredients can be materialized. We believe that this article provides an insight into the potential of bread waste and will encourage more researchers to contribute towards the goal of bread/bakery waste based biorefineries with the associated multiple benefits of minimizing/eliminating the enormous amount of this waste generated globally and creating wealth out of it.

## Availability of data and materials

Data sharing is not applicable to this article as no new data was created or analysed in this study.

## Conflicts of interest

There are no conflicts to declare.

## Supplementary Material
